# Marginal adaptation of customized gutta percha cone with calcium silicate based sealer versus MTA and biodentine apical plugs in simulated immature permanent teeth (an in vitro study)

**DOI:** 10.1186/s12903-024-04765-x

**Published:** 2024-09-11

**Authors:** Mary M. Mina, Sybel M. Moussa, Mahmoud R. Aboelseoud

**Affiliations:** Department of Conservative Dentistry, Faculty of Dentistry, Alexandria University, Champolion St., Azarita, Alexandria, 21527 Egypt

**Keywords:** Customized gutta percha, Calcium silicate, Mineral trioxide aggregate, Biodentine, Dental marginal adaptation, Scanning electron microscope

## Abstract

**Background:**

This study aimed to compare the marginal adaptation of a single customized gutta percha cone with calcium silicate-based sealer versus mineral trioxide aggregate (MTA) and Biodentine apical plugs in simulated immature permanent teeth.

**Methods:**

Thirty-nine extracted human maxillary anterior teeth were selected, prepared to simulate immature permanent teeth with an apical diameter 1.1 mm, placed in moist foam and divided into three groups. Group 1: Obturation with a single customized gutta percha cone and calcium silicate sealer. Group 2: MTA apical plug. Group 3: Biodentine apical plug. After incubation, teeth were horizontally sectioned at 1 mm and 3 mm from the apex and marginal adaptation was evaluated using scanning electron microscope (SEM).

**Results:**

Biodentine showed the least mean gap size at both 1 and 3 mm from the apex with no statistically significant differences compared to MTA (*p* > 0.05). The single customized cone with calcium silicate based sealer showed the greatest mean gap size at both 1 and 3 mm from the apex with a statistically significant difference compared to the other groups (*p* < 0.001).

**Conclusion:**

Biodentine and MTA apical plugs provide a significantly better marginal adaptation to the dentinal walls than a single customized gutta percha cone with calcium silicate based sealer in simulated immature permanent teeth.

## Introduction

A three-dimensional obturation with complete coronal and apical seal is critical for successful root canal treatment. This is essential to prevent bacteria and their byproducts, which remain after chemo-mechanical preparation of the root canal system, from reaching the apex resulting in apical periodontitis [1]. Additionally, it prevents fluid leakage from the periapical tissues which may provide a substrate for the remaining bacteria to flourish.

Root canal filling of immature permanent teeth requires particular management due to the technical difficulty to compact the root filling material against their open apices and thin dentinal walls [2]. These teeth often have irregular and wide apices, making conventional root canal filling inadequate for achieving a proper apical seal [3].

Regenerative endodontic treatment (RET) is a promising option for treating immature permanent teeth by replacing the damaged pulp with a viable tissue, allowing for continued root development and enhanced strength [4]. However, it is difficult to anticipate the result of RET [4, 5] which makes clinicians, even endodontists, hesitant to apply this treatment option [6]. Also, continued root development is still unpredictable [7]. Besides, this treatment option is restricted to compliant patients, teeth that do not need posts for restoration and it is more predictable in less infected teeth, teeth with larger apical sizes (> 1.1 mm) and younger patients [4].

Due to the limitations of RET, the apexification procedure has been widely used to manage these cases by inducing the formation of a calcific barrier. Calcium hydroxide was the first material used in apexification procedure. However, it requires a long time (6 to 24 months) to form an apical barrier, necessitating multiple visits and increasing the risk of fracture and reinfection of the root canal system. Also, the resulting barrier is porous and weak [8].

Mineral trioxide aggregate (MTA) has become the preferred material to be used as an apical plug in managing immature permanent teeth due to its osteoconductive properties, biocompatibility, resistance to dissolution in tissue fluids, and ability to enhance sealing by formation of interfacial deposits which fill the spaces and voids between the dentin and root filling material enhancing the frictional resistance [9, 10]. In addition, it has been shown to improve periapical healing by the formation of a new resistant bone in necrotic immature permanent teeth with apical periodontitis [11]. However, it has some drawbacks including prolonged setting time, discoloration, and poor handling [12].

Biodentine, a tricalcium silicate-based inorganic restorative cement, is claimed to be a bioactive dentin substitute with better biological and physical properties compared to MTA [13]. It offers a short setting time due to the presence of calcium chloride which acts as an accelerator and hydro-soluble polymer as a water-reducing agent [14]. It also has good handling and manipulation properties and has shown favorable results when used as an apical barrier in immature teeth which have necrotic pulps and wide-open apices [13].

Calcium silicate based (CaSi) sealers have been introduced and characterized by their good penetration into the dentinal tubules, interaction with the dentine moisture, and ability to form a secondary monoblock adhesion when used with gutta-percha for obturation, thereby strengthening the root structure [15]. They also form a mineral layer during their setting, inducing a chemical bond with the dentinal walls and enhancing their sealing ability [16]. Moreover, they can be used in conjunction with a single gutta-percha cone for obturation [17].

An alternative technique to properly seal the apical portion of a tooth with an open, irregular, or oval shaped apex is to customize a master gutta percha cone using different methods. Customization of gutta percha has been shown to significantly reduce the presence of voids within the apical 5 mm, reduce the extrusion of root filling materials and improve the replication of and adaptation to the canal wall [18]. The chloroform dip customization technique is highly effective in plasticizing gutta percha and chloroform is widely used in dental practice due to its fast action, low cost and simplicity in use. Although there are concerns related to its toxicity, previous research has considered chloroform to be safe for use in endodontics [19]. Moreover, applying it in small amounts within the root canals may prevent concerns about the risk of extrusion [20] and its continued use as a gutta-percha solvent is recommended if used carefully [21, 22].

Since the adaptation between the filling material and the root canal wall is crucial for minimizing microleakage, a customized gutta-percha cone tailored for each specific tooth might adapt better to the wide apical canal anatomy, especially when used with a CaSi sealer known for its bonding ability to dentine. Accordingly, the aim of this study was to evaluate and compare the marginal adaptation of a single customized gutta-percha cone (SCC) used with CaSi sealer versus MTA and Biodentine apical plugs in simulated immature permanent teeth. The null hypothesis was that there would be no significant difference in marginal adaptation to the root canal walls between the tested materials.

## Materials and methods

### Sample selection and preparation

**Fig. 1:**
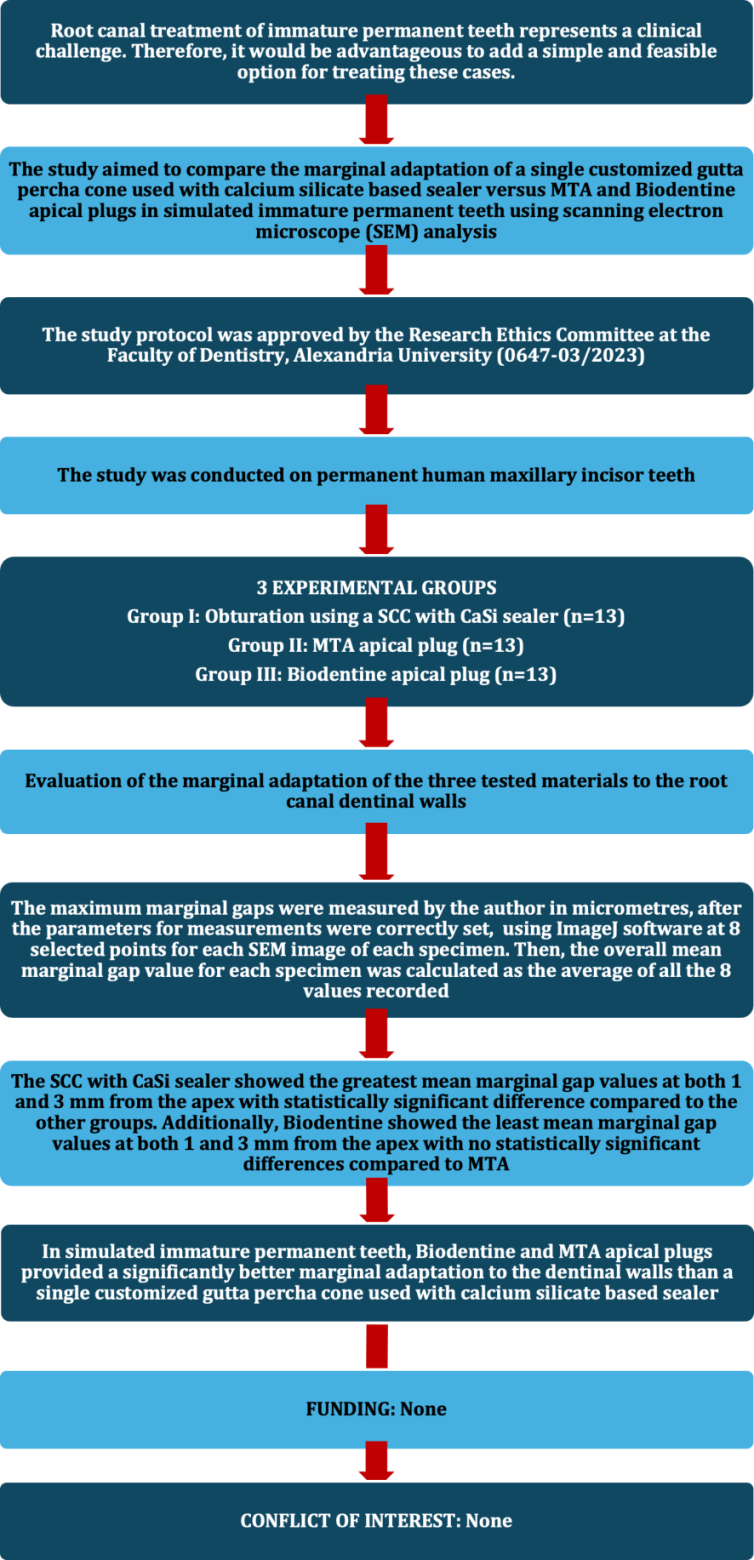
**PRILE 2021 flow chart for the current study**

This in vitro study followed the Preferred Reporting Items for Laboratory studies in Endodontology (PRILE) guidelines, 2021 [23]. The PRILE 2021 flowchart is presented in (Fig. 1).

The study protocol was approved by the Research Ethics Committee at the Faculty of Dentistry, Alexandria University (0647-03/2023). Sample size calculations were performed using MedCalc Statistical Software (Version 19.0.5).

Sample size was based on 95% confidence level to detect differences in the largest apical gap between Biodentine and MTA plug. De Sá et al. [24] reported median (range) of the largest apical gap = 29.86 (1.33–71.62) when MTA was used and 18.68 (5.94–55.07) when Biodentine was used. These estimates are equivalent to mean (SD) = 32.61 (22.72) and 23.60 (15.88), respectively. The calculated mean (SD) difference = 9.01 (19.30), 95% confidence interval= -9.41, 27.43. The minimum sample size was calculated to be 12 per group, increased to 13 to make up for laboratory processing errors. The total required sample size = number of groups × number per group = 3 × 13 = 39 [25].

To ensure adequate study power, post-hoc power analysis was performed, and the reported power ranged from 86 to 92% (G*Power Version 3.1.9.7).

In this study, 39 extracted human permanent maxillary anterior teeth with single straight roots, which were extracted for periodontal reasons, were collected from the Oral Surgery Department at the Faculty of Dentistry, Alexandria University, Egypt and examined to be free from cracks, fractures, curvatures, calcifications, resorption or developmental anomalies.

All teeth were immersed in 5.25% sodium hypochlorite for 30 min to remove soft tissue attached to the root surface and further cleaning was done by ultra-sonic scaler to remove any remaining calculus or soft tissue. After that, teeth were stored in saline solution to remain hydrated.

### Sample preparation to simulate immature permanent teeth

**Fig. 2:**
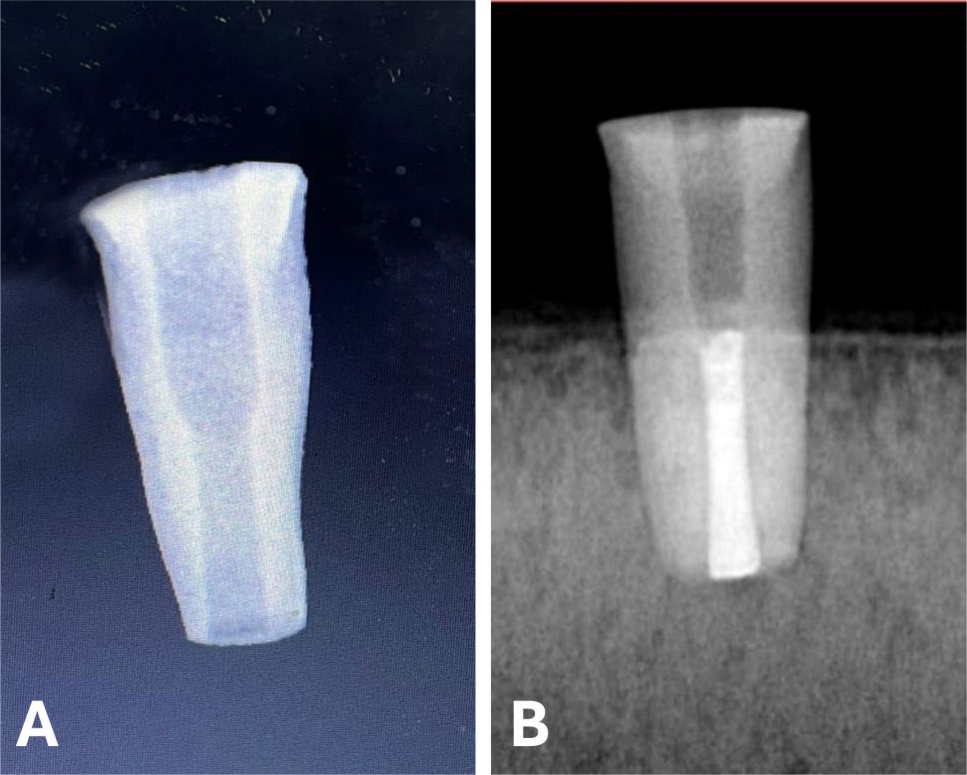
(**A**) Radiograph showing a specimen after being prepared to simulate an immature permanent tooth. (**B**) Radiograph showing a specimen after orthograde obturation with a calcium silicate based filling material

For each tooth, three mm of the root tip was removed [26] by a low-speed diamond saw, and the crown, together with the coronal portion of the root, was also sectioned by a low-speed diamond saw to standardize the root to 10 mm in length. A no.6 Peeso reamer (Mani, Tochigi, Japan) was used to drill from the coronal to the middle part of the root, and a no.3 Peeso reamer (Mani, Tochigi, Japan) was used to drill in a retrograde direction to create an open apex model as presented in (Fig. 2). This resulted in an apical diameter of 1.1 mm [27]. A large (#100-#110) k-file (Mani, Tochigi, Japan) was used gently on the canal walls 1 mm shorter than the working length to debride the canal walls and remove any irregularities.

Then, intra-canal irrigation was performed with 10 mL of 17% EDTA (MD.Cleanser, MetaBiomed, Korea) and the excess was removed with a high suction tip, followed by 10 mL of 5.25% NAOCL (Clorox for Chemical Industries, A.R.E) using a side vented needle to remove smear layer [28]. A final flush with 2 ml of sterile saline for 3 cycles, 20 s each, was used and the canal was dried with three paper points (Diadent, South korea) [29]. The samples were embedded in moistened floral arrangement foam to simulate the periapical environment and prevent the filling material extrusion out of the apex [27].

### Grouping

The specimens were numbered and randomly assigned into 3 equal groups (*n* = 13).

- **Group I**: Obturation using a single customized gutta Percha cone (Diadent, South korea) and calcium silicate sealer (NeoSEALER Flo; Avalon Biomed, Houston, TX, USA).
- **Group II**: MTA apical plug (NeoMTA – Avalon Biomed, Houston, TX – USA).
- **Group III**: Biodentine apical plug (Biodentine - Septodont – France).

### Teeth obturation

In group I: A gutta percha cone was selected to bind 1-mm short of the working length. A collagen sponge was placed apically by a hand plugger to avoid materials’ extrusion. The apical 2 mm of the gutta percha cone was dipped in chloroform (Al-Gomhoria chemical co. ltd, Egypt) for 1 s and the softened gutta percha cone was gently placed into a slightly moist canal, noting the orientation of the cone by marking the cone and tooth surface to be able to reinsert the cone at the exact same position at the time of obturation. The gutta percha cone was pushed to the canal terminus in a pumping motion and this was continued for another 10 s to allow the gutta percha to become firm without engaging undercuts [18]. Then, the premixed CaSi sealer was inserted by a syringe tip provided by the manufacturer 4 mm inside the root canal and the SCC was coated with the sealer and inserted in the root canal noting its correct orientation until it reaches the full working length. Finally, the cone was seared off at the level of the orifice. After obturation, mesiodistal and buccolingual digital radiographic images of the root were taken with sensor size 1 (Waldent Carpo RVG V-Sensor) using x-ray machine (EzRay Air Portable, Vatech) with the following parameters: 65 kVp, 2.5 mA, 0.5 s exposure time and 0.4 mm focal spot to assess obturation quality.

In groups II and III: MTA and Biodentine were manipulated according to the manufacturer’s recommendation and were delivered to the root canal by an MTA carrier after placement of a collagen sponge to prevent material extrusion. Material condensation was done with appropriate hand pluggers to form 5 mm of orthograde MTA and Biodentine plugs. The quality and thickness of the apical plugs and the coronal space were checked by mesiodistal and buccolingual digital radiographs. A moist paper point was then placed into each root canal followed by placement of a temporary restorative material (Cavit, 3 M ESPE, St. Paul, MN, USA).

Specimens were then incubated at a temperature of 37 °C and 100% relative humidity for 7 days allowing complete setting of the used filling materials [30].

All specimens were prepared and filled by the same operator.

### Scanning electron microscope analysis

**Fig. 3:**
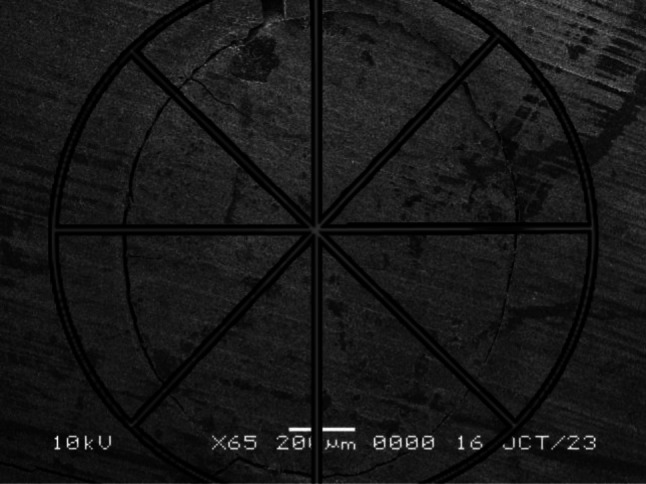
SEM micrograph clarifying the methodology of measurements. Each transverse section was divided into 8 parts and the maximum marginal gap value of each part was recorded using ImageJ software. The overall maximum marginal gap value was calculated as the average of all 8 values recorded for each specimen

Each tooth was mounted on an acrylic block and was then transversally sectioned at one and three mm from the resected apex using a microtome (MICRACUT 150, Kemet International, UK). All specimens were sputter coated with gold–palladium and viewed with a scanning electron microscope (JEOL JSM-5510LV, Tokyo, Japan). The marginal gap was measured using ImageJ software (Wayne Rasband; National Institute of Health, Bethesda, MD, USA) following the methodology proposed by Shokouhinejad et al. [31]. To measure the marginal gap, micrographs obtained from the SEM were digitally divided into eight slices for each transverse section. The marginal gaps were measured using the freehand and the line selection tools of ImageJ software at the 8 selected points for the measurements. This was done after the parameters for measurements were correctly set using the set scale and the scale bars of the pictures. For each slice, the maximum gap value in terms of distance (µm) between the root canal wall and the filling material was recorded, and for each specimen the overall maximum gap value was calculated by calculating the average of all the eight slice values recorded (Fig. 3).

These steps were repeated for each section for all specimens [32].

Training and calibration on the assessment method was performed for a single examiner, intra-examiner reliability was calculated, and Intraclass Correlation Coefficient (ICC) ranged from 0.86 to 0.97 indicating excellent reliability [33].

### Statistical analysis

Normality was tested using descriptive statistics, plots (histogram and Q-Q plot), and Shapiro Wilk normality test. Data showed non-normal distribution, so non-parametric analysis was adopted. Descriptive statistics were calculated as means, medians, standard deviation (SD), and interquartile range (IQR). Comparisons between the three study groups were performed using Kruskal Wallis test, followed by multiple pairwise comparisons using Bonferroni adjusted significance level. Comparisons of the marginal gap at 1- and 3-mm within each group were performed using Wilcoxon signed ranks test. Significance was set at p value < 0.05. Data were analyzed using IBM SPSS for Windows (Version 26.0).

## Results

**Fig. 4:**
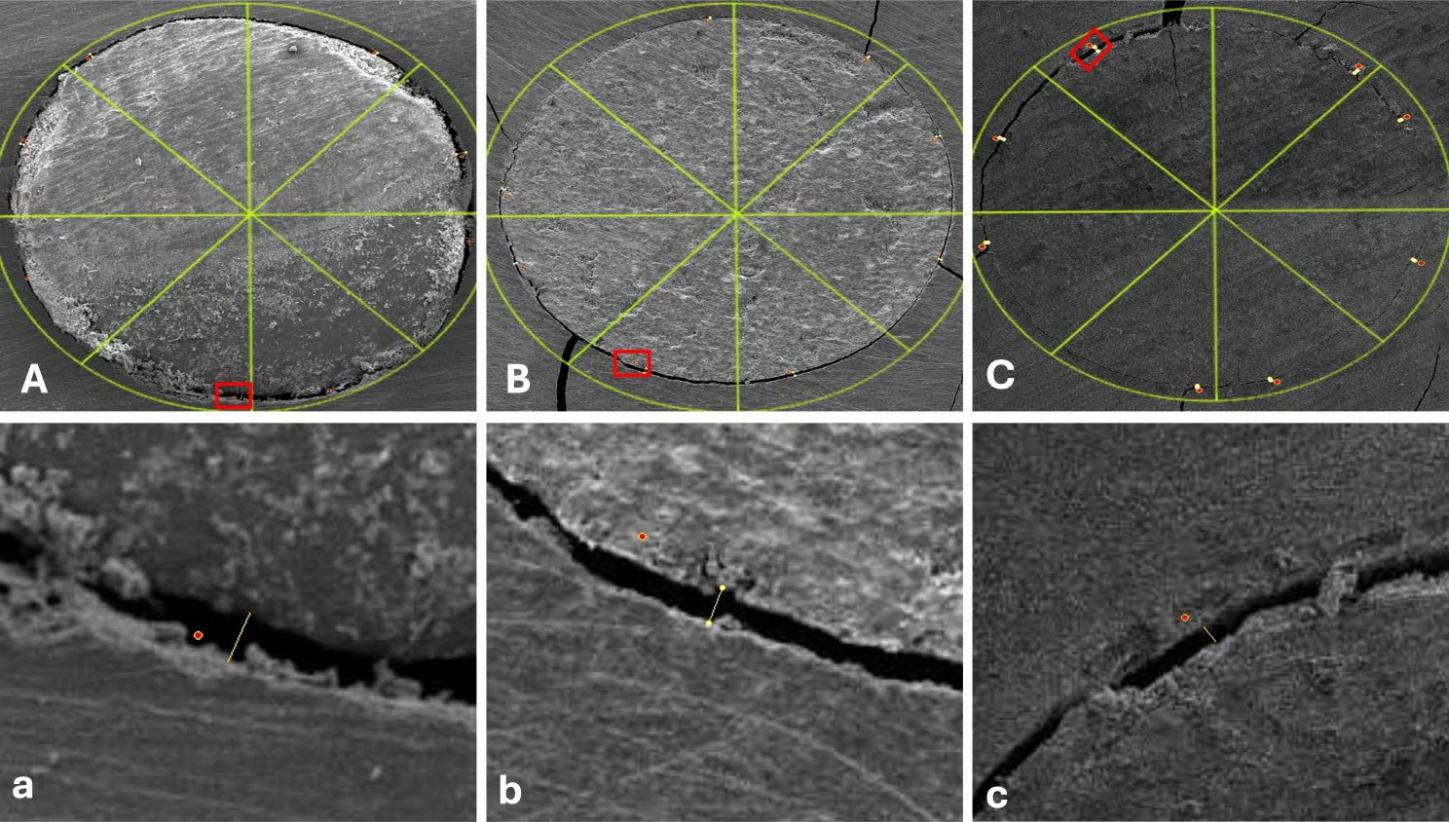
Representative SEM micrographs showing the marginal adaptation of the three tested techniques circumferentially at 1 mm from the apex (images **A**, **B**, **C**) and the maximum gap value recorded at the selected sites (red boxes) (images **a**, **b**, **c**). (**A**, **a**) SCC with CaSi sealer. (**B**, **b**) MTA apical plug. (**C**, **c**) Biodentine apical plug

**Fig. 5:**
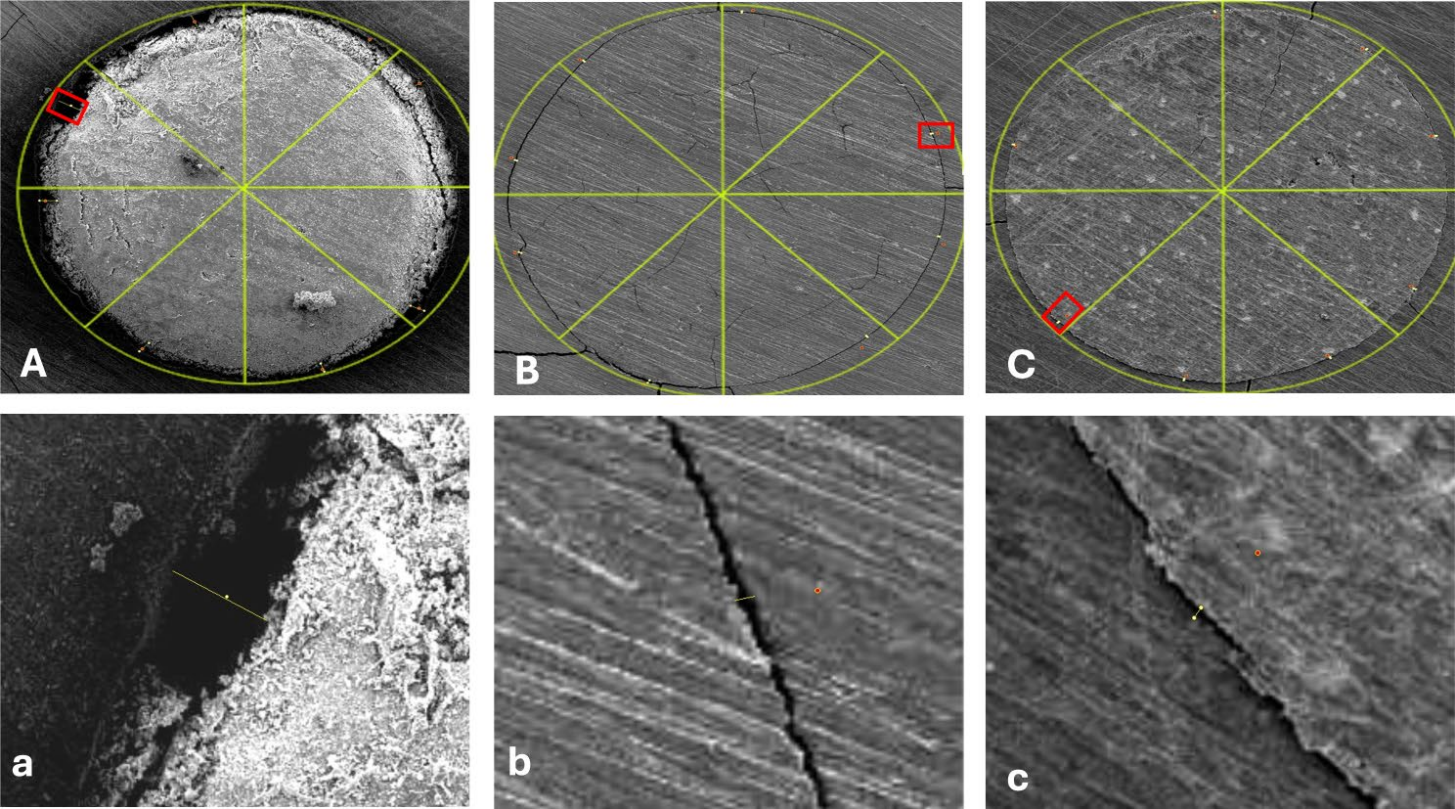
Representative SEM micrographs showing the marginal adaptation of the three tested techniques circumferentially at 3 mm from the apex (images **A**, **B**, **C**) and the maximum gap value recorded at the selected sites (red boxes) (images **a**, **b**, **c**). (**A**, **a**) SCC with CaSi sealer. (**B**, **b**) MTA apical plug. (**C**, **c**) Biodentine apical plug

A statistically significant difference was found between group I (SCC with CaSi sealer) and the other two groups (P value **< 0.001)** at both 1 mm from the apex (mean gap size = 25.91 ± 14.55) and at 3 mm from the apex (mean gap size = 29.44 ± 11.76). This group revealed the largest mean gap size measurements with the least marginal adaptation. (Figures 4 and 5)

In group II (MTA), the mean gap size was (6.98 ± 2.8) at 1 mm and (4.85 ± 1.4) at 3 mm.

**Fig. 6:**
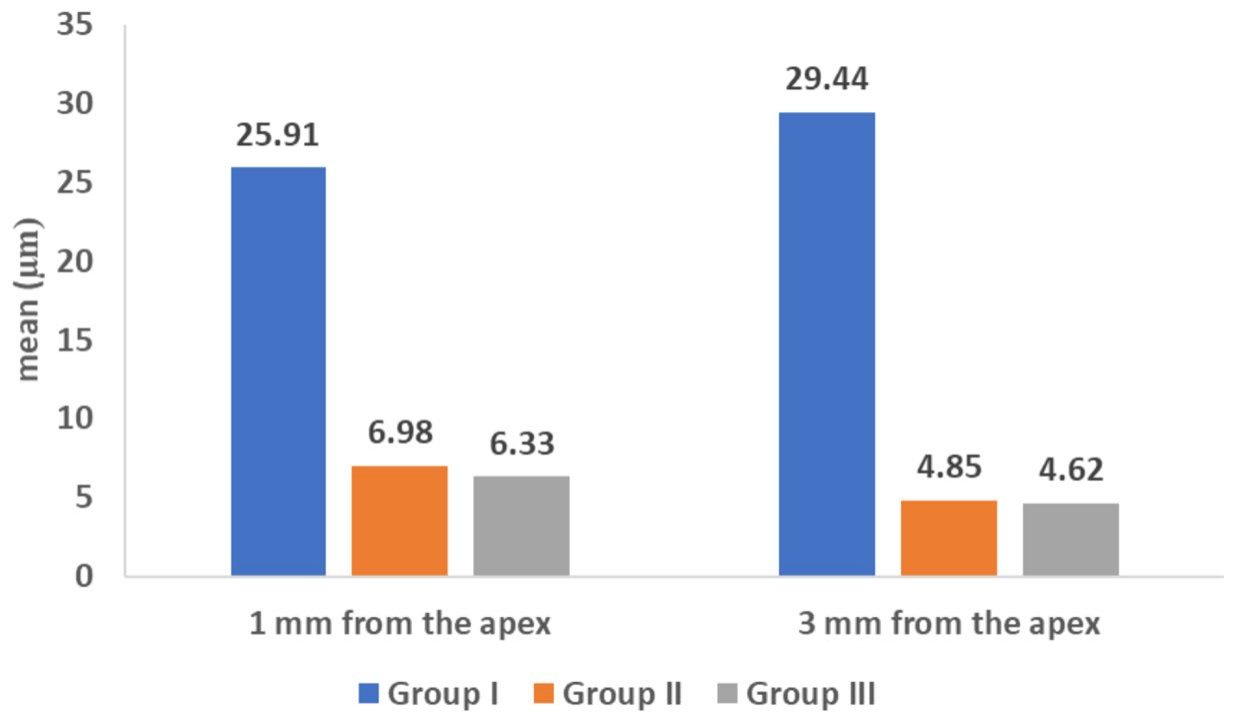
Mean marginal gap (in µm) in the three study groups

In group III (Biodentine), the mean gap size was found to be (6.33 ± 1.3) at 1 mm and (4.62 ± 1.47) at 3 mm, which was the least mean gap size in all groups (Fig. 6). However, there was no statistically significant difference between MTA and Biodentine when comparing them at both 1 mm and 3 mm sections.

When comparing the mean gap size between 1 and 3 mm from the apex within each study group, no statistically significant difference was noted in group I (P value = 0.65) while a significant difference was found in groups II and III (P value = 0.02, 0.002 respectively) in which the mean gap size was greater at 1 mm than at 3 mm from the apex in both groups (See Table 1.

**Table 1:** Comparison of the mean marginal gap (in µm) between the three study groups

		Group 1 (SCC with CaSi sealer)	Group 2 (MTA apical plug)	Group 3 (Biodentine apical plug)	*P* value 1
1 mm	Mean (SD)	25.91 (14.55) **a**	6.98 (2.80) **b**	6.33 (1.30) **b**	< 0.001*
	Median (IQR)	23.59 (14.21, 34.82)	6.16 (4.77, 8.18)	6.11 (5.85, 7.06)	
3 mm	Mean (SD)	29.44 (11.76) **a**	4.85 (1.40) **b**	4.62 (1.47) **b**	< 0.001*
	Median (IQR)	29.23 (20.69, 35.58)	4.61 (3.94, 5.35)	4.57 (4.04, 5.11)	
P value 2		0.65	0.02*	0.002*	

SD: Standard deviation, IQR: Interquartile range

P value 1: Kruskal Wallis test

P value 2: Wilcoxon signed ranks test

*Statistically significant at p value < 0.05

a-b: small letters denote significant differences between different groups at 1 mm and 3 mm from the apex using Bonferroni adjustment

## Discussion

Several studies have shown a correlation between apical microleakage rates and marginal gaps between the filling materials and dentinal walls of the root canals [34, 35]. The rationale of our study was to test the ability of the SCC with CaSi sealer to achieve a comparable marginal adaptation to MTA and Biodentine apical plugs which might add another simple and feasible treatment modality for management of immature permanent teeth.

In the present study, SEM analysis showed a statistically significant difference between the SCC with CaSi sealer and the other groups. Therefore, the null hypothesis was rejected.

Chloroform dip technique was chosen to customize the master cone in the current study because chloroform was reported to have the strongest dissolving or softening activity for gutta percha points compared to other alternatives [36].

It has been reported in many studies that CaSi sealers are biocompatible with antimicrobial properties and are bioactive which can stimulate hard tissue formation [37]. Accordingly, their biocompatibility can make them suitable to be used in cases of immature apices in which unintended extrusion of material might happen. Additionally, they exhibit advantageous penetration of the dentinal tubules which provides an increased contact surface between the filling material and dentinal walls [38] and can also form a chemical bond to dentin, resulting in an improved sealing ability [39]. A novel CaSi sealer (NeoSEALER Flo, Avalon biomed) was used in the current study as it has been reported to have a high push out bond strength in the apical third [40] and according to the manufacturer, it is bioactive, promotes hydroxyapatite formation, resin free, non-staining, biocompatible and has superior handling properties.

Extracted maxillary anterior human teeth (central and lateral incisors) were chosen in this study due to their relatively uniform canals with less variation in their root canal anatomy and 3 mm were sectioned apically to exclude curvature, lateral canals which helped in standardization of the specimens. In addition, they are the most affected teeth with pulp necrosis and immature apices due to trauma [41].

Open apex teeth models were prepared in a similar way to that used by Lertmalapong et al. [27] but a Peeso reamer size 3 was used in our study in a retrograde direction, instead of size 4 used in the mentioned study, to standardize apical diameter to 1.1 mm. This diameter was chosen because it is large enough to be difficult to achieve a good seal with the conventional obturation method resembling stage 9 in Nolla’s classification of tooth development [42].

Clinical environment was simulated by placing the prepared roots in moist floral arrangement. This was beneficial in providing hydration to the roots which was needed for the setting of the calcium silicate materials used and limiting the filling materials extrusion from the apex. Moreover, a collagen sponge was placed apically against which the filling materials were packed, minimizing their iatrogenic extrusion.

In the current study, the smear layer removal was done using 10 ml of 17% EDTA followed by 10 ml of 5.25% NaOCl with a final flush of sterile saline to avoid the adverse effects of EDTA as it can disrupt the hydration of calcium silicates decreasing their hardness and biocompatibility due to calcium chelation [37]. Moreover, the canals were left moist by using 3 paper points only to allow setting of the used materials which require hydration to set as it was shown in a previous study that slightly moist canal walls resulted in the highest push out bond strength for the tested CaSi sealer [43].

Although there is controversy related to the proper thickness of the apical plug used for apexification of immature permanent teeth, many researchers have agreed that 5 mm thickness could be adequate to provide a good apical seal [44], which was applied in our study.

It is important to mention that although the three tested groups in the current study showed good obturation quality in both mesiodistal and buccolingual digital radiographs, SEM analysis revealed marginal gaps. SEM analysis was chosen as a method of evaluating marginal adaptation of the filling materials tested due to its high resolution, enhanced interface magnification, and superior depth of field [45]. The maximum gap between the filling material and the dentinal walls of the root canal was measured in each specimen as a mean of 8 measurements circumferentially to be more accurate as the gaps are assessed throughout the whole perimeter of the root canal.

The results of our study differ from a study by Hamdan et al. [46] who concluded that there was no difference in leakage resistance between a customized gutta percha cone with BioRoot sealer and MTA apical plug in immature permanent teeth as they both showed unsatisfactory apical seal and nearly all specimens showed dye penetration. This contradiction may be due to different methodology and testing tools in which dye penetration test was used in the mentioned study to assess the microleakage. However, dye penetration test was avoided in our study because it has been reported to have questionable reliability and clinical relevance [47].

A possible reason for the maximum marginal gaps found in the SCC with CaSi sealer group is that two materials are used in obturation unlike MTA or Biodentine which are packed and condensed as one obturation material and provided good apical seal and smaller marginal gaps.

The marginal gaps were larger at 3 mm from the apex than at 1 mm in the SCC with CaSi group, although there was no significant difference statistically, this may be due to insufficient softening of the customized cone in which only the most apical 2 mm were dipped in chloroform for one second following a technique used in a previous study by Van Zyl et al. [18] but this might not be enough to take a proper impression of the large canals in the current study especially at 3 mm from the apex noting that the previous study was done on mature teeth with smaller root canals and apices.

Biodentine apical plug showed the best marginal adaptation in the current study with the least mean gap at its interface with the dentinal walls, followed by MTA with no significant difference between them. This is in line with the results obtained by de Sá et al. [24] who concluded that there was no significant difference between the marginal adaptation of MTA and Biodentine to dentinal walls when used as apical plugs in simulated immature teeth and is also in agreement with Bani et al. [10] who used fluid filtration technique and concluded that the sealing ability of Biodentine and MTA were similar at any apical plug thickness. Moreover, Abbas et al. [48] showed that both materials had the same sealing ability using bacterial leakage method of assessment. However, in a study by Refaei et al. [49], Biodentine resulted in significantly less microleakage than ProRoot MTA when used as orthograde apical plug in immature permanent teeth.

Interestingly, the marginal adaptation of both Biodentine and MTA at 3 mm from the apex was significantly better than at 1 mm. This could be explained by the better packing of the material against the previously placed layers at 3 mm than against the less firm collagen sponge in the first layer placed at 1 mm from the apex.

It is worth mentioning that the current study has some limitations. First, one method of assessment was used to test the sealing ability of the used materials, however, it is suggested to combine two or more methods of microleakage assessment to give more reliable results [47]. Second, the study is limited to the straight canals of maxillary anterior teeth in which the placement of apical plugs is easier and more predictable than in curved canals or those with difficult clinical accessibility. Third, the sample size isn’t large enough to generalize the results.

Based on the results of the current study, it is recommended to further evaluate the customized gutta percha cone technique using other types of solvents such as orange oil, eucalyptol, or using heat, customizing gutta percha cone with longer dipping time or dipping a larger area in the solvent. Furthermore, other types of CaSi sealers or a resin sealer could be tested with the customized gutta percha cone. In addition, the apical sealing ability of a SCC with CaSi sealer and warm vertical compaction should also be evaluated. Finally, further studies with larger sample sizes are recommended for more accuracy.

## Conclusions

Within the limitations of this study, it can be concluded that the marginal adaptation of Biodentine and MTA apical plugs to the dentinal walls of the root canals are significantly better than that of a customized gutta percha cone used with calcium silicate-based sealer in immature permanent teeth models.

## Data Availability

All data included in this study are available from the corresponding author upon request.
